# Therapeutic Approaches for Age-Related Macular Degeneration

**DOI:** 10.3390/ijms231911769

**Published:** 2022-10-04

**Authors:** Ruth M. Galindo-Camacho, Cristina Blanco-Llamero, Raquel da Ana, Mayra A. Fuertes, Francisco J. Señoráns, Amélia M. Silva, María L. García, Eliana B. Souto

**Affiliations:** 1 Department of Pharmaceutical Technology, Faculty of Pharmacy, University of Porto, Rua de Jorge Viterbo Ferreira, 228, 4050-313 Porto, Portugal; 2 Department of Pharmacy and Pharmaceutical Technology, and Physical Chemistry, Faculty of Pharmacy and Food Sciences, University of Barcelona, 08028 Barcelona, Spain; 3 Unit of Synthesis and Biomedical Applications of Peptides, IQAC-CSIC, 08034 Barcelona, Spain; 4 Institute of Nanoscience and Nanotechnology (IN2UB), University of Barcelona, 08028 Barcelona, Spain; 5 Healthy Lipids Group, Departmental Section of Food Sciences, Faculty of Sciences, Autonomous University of Madrid, 28049 Madrid, Spain; 6 Department of Biology and Environment, University of Trás-os-Montes e Alto Douro, UTAD, Quinta de Prados, 5001-801 Vila Real, Portugal; 7 Centre for Research and Technology of Agro-Environmental and Biological Sciences, CITAB, UTAD, Quinta de Prados, 5001-801 Vila Real, Portugal; 8 REQUIMTE/UCIBIO, Faculty of Pharmacy, University of Porto, Rua de Jorge Viterbo Ferreira, 228, 4050-313 Porto, Portugal

**Keywords:** age-related macular degeneration, vascular endothelial growth factor, nanocarriers, drusen, retinal pigment epithelium, 3D bioprinting

## Abstract

Damage to the retinal pigment epithelium, Bruch’s membrane and/or tissues underlying macula is known to increase the risk of age-related macular degeneration (AMD). AMD is commonly categorized in two distinct types, namely, the nonexudative (dry form) and the exudative (wet form). Currently, there is no ideal treatment available for AMD. Recommended standard treatments are based on the use of vascular endothelial growth factor (VEGF), with the disadvantage of requiring repeated intravitreal injections which hinder patient’s compliance to the therapy. In recent years, several synthetic and natural active compounds have been proposed as innovative therapeutic strategies against this disease. There is a growing interest in the development of formulations based on nanotechnology because of its important role in the management of posterior eye segment disorders, without the use of intravitreal injections, and furthermore, with the potential to prolong drug release and thus reduce adverse effects. In the same way, 3D bioprinting constitutes an alternative to regeneration therapies for the human retina to restore its functions. The application of 3D bioprinting may change the current and future perspectives of the treatment of patients with AMD, especially those who do not respond to conventional treatment. To monitor the progress of AMD treatment and disease, retinal images are used. In this work, we revised the recent challenges encountered in the treatment of different forms of AMD, innovative nanoformulations, 3D bioprinting, and techniques to monitor the progress.

## 1. Introduction

Age-related macular degeneration (AMD) is a common multifactorial eye disease of the central area in the ocular posterior segment, also known as *macula lutea* [1,2]. This is an essential retinal area for the vision of fine details and image resolution by capturing the greatest focus of external light stimuli [1]. AMD affects the macular region of the retina, leading to progressive loss of central vision [3], with the consequent impact on both patients and society. It is a complex disease, resulting from the interaction of diverse factors [4], including infection [5], and is the main cause of irreversible blindness in patients over 65 years old. The literature reports that there are about 170 million people in the world affected by AMD, with it being the third leading cause of blindness worldwide, corresponding to about 9% of these cases. AMD prevalence is likely to increase up to 288 million by 2040, as a consequence of the exponential aging of populations worldwide [6]. There are numerous heterogeneous risk factors linked to this disease, with ageing being the main one. In addition to aging, environment and lifestyle, together with the genetic background and para-inflammation, have also been associated with AMD pathogenesis [7]. Race seems to be another relevant risk factor, as the prevalence of any form of AMD, in an early or late stage, is reported to be higher in Europeans than Asians (16% and 9%, respectively). A metadata analysis by Zhou et al. (2021) also showed a greater incidence of both early and late AMD among European descendants [8]. Moreover, when considering exudative and nonexudative form, Caucasian people have much higher risk to develop wet AMD and vision loss when compared with black people and Hispanics, and they also show higher incidence of nonexudative AMD than African Americans, namely in Caucasian patients with light-colored eyes. Although it is clear that ethnic differences are a major risk factor for AMD development, its mechanisms are still not well explained. However, genetic factors appear to have a greater influence on risk than diet [2,9]. Smoking is a modifiable factor associated with a two-fold increased risk for the onset of AMD, due to the induction of oxidative stress and antioxidant depletion, both associated with retinal damage. Other modifiable factors, such as higher body mass index (BMI), are found to be associated with increased risk of developing AMD because of pro-inflammatory factors, such as complement components and cytokines, are elevated in obese individuals, disturbing the functions of the RPE whereas the accumulation of carotenoids in adipose tissue reduces their availability in the macula [7]. Diet was also reported to play a significant role in the risk development of AMD. The literature describes that a fat-rich diet, namely trans-fat, is responsible for a higher risk of developing AMD. Phototoxic exposure also strongly contributes to significantly increasing AMD risk together with several gene polymorphisms [1,2]. 

Patients with AMD suffer a great decrease in their life quality because many daily activities, such as driving and reading, require functional central visual perception. Moreover, these patients are often diagnosed with depression and present frequent bones fractures because of recurrent falls attributed to vision loss [2]. 

Considering the normal retinal architecture, *macula lutea* is a central, posterior portion of the retina. This structure has the highest cone photoreceptors’ concentration within the retina, which are responsible for central high-resolution visual acuity, allowing the visualization of fine details, as well as color vision. Retinal Pigment Epithelium (RPE) is part of the outer blood retinal barrier composed of a single layer of polygona cells, arranged at the external layer of the sensory retina. It is located between the Bruch’s membrane and the outer segment of photoreceptor cells. The main functions of RPE include light absorption, phagocytosis, nutrient transport, and cytokine secretion [10]. REP dysfunction and damage play a crucial role in the pathogenesis of AMD, although perturbations in other tissues such as choroid, Bruch’s membrane, and photoreceptors are also critical factors [11,12]. 

This damage can lead to a drusen development between Bruch’s membrane and the retinal pigment epithelium, that may initiate several pathogenic processes leading to blindness [13]. Drusen are composed of lipids (such as phospholipids, triglycerides, cholesterol, cholesteryl esters), vitronectin, apolipoproteins, and inflammatory and amyloid proteins that create, focal deposits of extracellular debris, with pale and yellow aspect [2], resulting in lesions in both macula and peripheral retina [10]. Depending on the number, size and shape, drusen is reported to be a manifestation of either normal ageing or an early sign of the onset of AMD [14]. The classification grade of AMD is based on the categorization of drusen, considering their size and the appearance of their margins as hard or soft [10].

With ageing, the retinal innate immune system-like microglia and the complement system shows low levels of activation because of the chronic low grade in the oxidative stress levels. This phenomenon is called para-inflammation and its response allows the maintenance of homeostasis in older healthy eyes. This response is dysregulated in patients with AMD, which promotes the onset of macular damage. The chronic inflammation related to para-inflammation dysregulation is influenced by the above-mentioned risk factors (e.g., genetic predisposition, age, and behavioral habits). The literature reports that it is believed that oxidative stress and light may be the main trigger of AMD, by combining the exposure of the retina to oxygen and light [2,13]. While the appearance of large drusen and focal hyperpigmentation have been strongly linked to the aging process, the Waterman study also supports the disappearance of non-neovascular features of AMD and may be instrumental to complement independent gradings of the disease [15].

Damage and malformation of cells and tissue occur mainly because of accumulative oxidative and metabolic changes induced by reactive oxygen species [11]. Age-related degeneration is caused by the imbalance between tissue damage induced by ROS and the repair/remodeling process, which is a fundamental process for AMD. In fact, the main risk factors increase ROS generation. The innate immune system plays a key role, not only in the tissue reparation process, but also in AMD susceptibility. The external complex formed by RPE and photoreceptors, which is the site of elementary AMD lesions, is regarded to be more sensitive to oxidative stress, considering the proximity with high variable choroidal hemodynamics and the light stimuli because of the continuous exposure to photooxidation. Photooxidation of photoreceptors is associated with the complement activation, leading to complex formation, which is a trigger to apoptotic processes resulting in retinal degeneration [1]. Pro-inflammatory cytokines (IL-6, IL-8, and TNF-α) are macrophage stimulators, and by the stimulation of the tissue complement system, the homeostasis of tissue repair is maintained, and the functionality is restored. These tissue factors are released into the circulation and stimulate the systemic immune system. Innate immune response by complement pathway is initiated through tissue stress to promote tissue repair. This response, which helps the adaptation of tissue under stressful conditions leading to the functionality restoring, is the para-inflammation [2].

**Figure 1:**
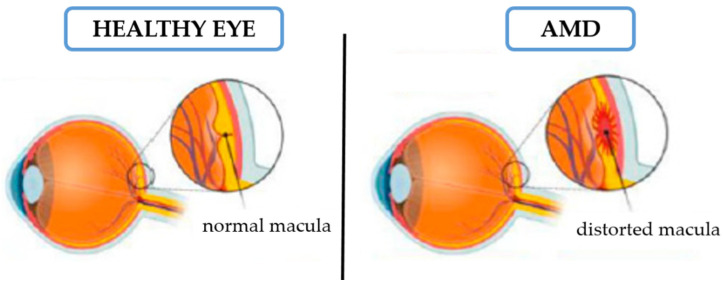
Appearance of macula in a healthy (**left**) and in a damaged (**right**) eye (reproduced from Fernandes et al. (2022) [2], CC BY license).

Although traditionally AMD has been classified into two clinical sub-types (Figure 1): dry (non-exudative or non-vascular) and wet (exudative or neovascular), based on the presence or absence of new blood vessels [2], significant overlap exists in the underlying pathogenic mechanisms of both clinical conditions [12]. Some differences between both can be pointed out. 

For dry AMD, based on the accumulation of extracellular material under RPE, the atrophic and hypertrophic alterations occur in the epithelium underlying the central macula, promoting the loss of photoreceptors. In this type of AMD, because of drusen deposition, the RPM cells suffer degeneration and atrophy, leading to central vision lost. AMD in its early stage can include clinical signals, such as drusen and irregularities in the retinal pigment epithelium. Nonexudative AMD can progress to the exudative form of AMD [2], with vascular endothelial growth factor (VEGF) being the key mediator in its progression). In wet AMD form, new vessels grow from the choroid through Bruch’s membrane with proliferation above or below RPE, under the retina in the macular region. In this way, pathological choroidal neovascular membranes (CNM) are formed and they can potentially lead to edema, where extravasation of blood and lipid materials is seen, due to the leaky properties of the newly formed vessels. The most dangerous form of CNM leads to a dense, misshapen fibrovascular scar that affects central vision for a short time, if not treated properly [2,13].

AMD involves the use of a wide vocabulary for its definition and classification, taking into account the signs and symptoms that patients develop. In addition, diagnostic imaging analysis, and the lessons learnt from therapeutic results, have changed substantially over the years. Spaide et al. (2020) [12] proposed a consensus nomenclature system to define AMD and frames the subtypes of neurovascular AMD, in order to facilitate the use of a uniform set of terminology in clinical practice [12]. 

## 2. AMD Signs and Symptoms

The progression of AMD happens over an extended time frame. In the early stages, AMD is often an asymptomatic disease, and characterized by the presence of medium size drusen in the macula. Intermediate dry AMB is regarded as the presence of large drusen (>125 mm), RPE abnormalities and alterations in photoreceptors or Bruch’s membrane. Visual symptoms remain mild, ranging from no symptoms to mild blurring, metamorphopsia and scotoma. Geographic atrophy (also known as the non-neovascular advanced stage of dry atrophic) AMD is associated with the degeneration of RPE, the retina and choriocapillaris [16].

This geographic atrophy can be unifocal or multifocal, and can also surround and spare the central macula. Its progression could be detected and monitored by imaging techniques. The symptoms include mild central distortion, which occurs when patients are reading, driving, or watching television, with it also being difficult for the patient to recognize faces.

Neovascular AMD incorporates some typical lesions, such as the presence of fluid or retinal hemorrhage (intraretinal, subretinal, or retinal pigment epithelium), retinal pigment epithelial detachment, exudate, and subretinal fibrous scar tissue. These manifestations are easy to detect by imaging, which also provides information about the size, location, and extent of drusen, as well as the presence and activity of choroidal neovascularization

With age, other alterations occur, namely changes in the retina, such as RPE cellular size and shape, thickening of Bruch’s membrane, thickening of the internal limiting membrane, and the decrease of retinal neuronal elements [17]. The progression of wet AMD is triggered by an inflammatory cascade. It is activated by the drusen formation since it contains glycoproteins and lipids, and inflammatory components from the cascade pathway that cause the loss of photoreceptors [2,17]. Loss of function of the Bruch’s membrane is a crucial point in this process that occurs as a consequence of RPE degeneration, once changes in the permeability of Bruch’s membrane lead to the deposition of the extruded material that are responsible for drusen development. When its integrity is broken, a process of choroidal neovascularization (CNV) is observed, whereby neovascular complexes from the choroid grow into the sub-pigment epithelial and subretinal spaces. The Bruch’s membrane failure is associated with the increase in vascular endothelial growth factors (VEGF) leads to the growth of atypical choroidal vessels under RPE and also under the retina. These newly formed vessels can lead to bleeding because of scar formation. Excessive growth of abnormal blood vessels is a process observed in AMD corresponding to the CNV process and in which the VEGF subfamily plays a critical role. In the most severe form of wet AMD, there is a development of a disciform spot in the macula that leads to permanent and irreversible loss of central vision. Platelet-derived growth factor (PDGF) is a critical factor for pericytes maintenance. It is crucial for new vessels’ formation and pericytes action control, as well as their growth and viability to prevent capillary malformation and retinal neovascularization. VEGF is composed of diverse families that play different roles. VEGF-A is a critical factor in neovascularization, and thus the main target of anti-VEGF drugs. Ocular levels of VEGF are low under normal physiological conditions, but they are very high in the affected areas when under pathological circumstances. High levels of VEGF in RPE stimulate the barrier integrity breakdown and promote neovascularization. It is still important to determine how the risk of AMD is increased by aging and how significant age-related vascular dysfunction contributes to AMD progression. Since this physiological barrier of endothelial cells regulates vascular functions, endothelial dysfunction in the choriocapillaris plays an important role in AMD pathogenesis [2]. 

Although there are pharmacological treatments for wet AMD available and there are different types of drugs that are being proposed for the management of the disease, there is no ideal treatment available for AMD yet. However, the gold standard treatment proposed for patients with AMD is based on limiting the function of the vascular endothelial growth factor (VEGF) [18,19,20] via intravitreal injection of anti-VEGF molecules, which were found to be effective in avoiding the loss of visual function and stabilizing disease progression [18,21,22,23,24,25,26,27]. There are currently only three drugs that contain anti-VEGF molecules approved by the United States Food and Drug Administration (FDA) for the treatment of AMD. Table 1 lists the available synthetic drugs used in clinic, describing their structure and their mode of action (MOA). Only Brolucizumab^®^, Aflibercept^®^ and Ranibizumab^®^ are approved by the FDA. Ranibizumab^®^ is a humanized monoclonal antibody fragment which targets VEGF-A, Brolucizumab^®^ is a single chain fragment of humanized anti-VEGF-A antibody, and Aflibercept^®^ is a soluble protein which targets VEGF receptor (VEGFR). All drugs have shown protective effects in AMD patients, although recent studies demonstrate that Brolucizumab^®^ has better safety and efficacy profiles than Aflibercept^®^ [22], attributed to Brolucizumab^®^’s ability to cross retinal layers leading to higher exposure in the retina and RPE/choroid [26]. On the other hand, recent studies report that Ranibizumab^®^ 0.5 mg treatment (clinical dosage in use) for 12 months improved visual outcomes in Taiwanese patients with AMD [28], with the drug being the one with the lowest dose required for the treatment (Table 1) 1. Faricimab^®^ is a recently approved angiopoietin-2 and VEGF-A inhibitor showing promising results in extending time intervals between ocular injections. Conbercept^®^ binds to PLGF, VEGF-A, and VEGF-B and C and is approved for use in China. 

Despite the wide recommendation and clinical use of anti-VEGF treatment for AMD patients, the main reported disadvantage associated with this therapy is mainly related to the short half-life of the anti-VEGF molecules, and the consequent demand for repeated injections [20,21]. With respect to dry AMD, there are no currently available treatments. However, ongoing trials are underway to explore newer agents in the treatment and prevention of AMD with other therapeutic targets. For example, L-DOPA has been investigated as an AMD treatment. In these studies, exogenous L-DOPA protected against AMD. In fact, L-DOPA is normally produced in pigmented tissues, such as the retinal pigment epithelium, as a byproduct of melanin synthesis by tyrosinase [29,30]. Alternatively, clinical trials studying stem cell therapy based on the transplantation of RPE cells for tissue damaged by CNV or tissue loss owing to geographic atrophy are underway [18,31,32]. Some of the supplements based on natural drugs and advised in patients with early AMD include InShape^®^, Nutrof^®^, Ocuvite^®^, and Vitalux^®^ [33]. Regarding natural drugs proposed for the early-stage prevention of AMD, most of them are based on the oxidative stress as the main therapeutic target for the management of the disease [23]. The degeneration of RPE cells lead to the progression of AMD. This influences lipofuscin and drusen accumulation, and thus, the development of chronic inflammation. Potential future therapeutic strategies for the prevention and treatment of AMD should focus on the suppression of oxidative stress and inflammation [34]. The carotenoids lutein and zeaxanthin, and Omega-3 lipids are the natural compounds with the major proven efficiency in the reduction of the progression of AMD and are described as alternative potential drugs for early AMD patients [18,27,31,32,35,36]. Docosahexaenoic acid (DHA) and eicosapentaenoic acid (EPA, the precursor to DHA) can be obtained from fish, krill, and microalgae. Vital retinal functions depend on the existence of an adequate proportion of DHA in retinal lipids. DHA intake and Omega-3 LC-PUFAs can influence AMD by inhibiting choroidal neovascularization, triglyceride lipoproteins interact with retinal pigment epithelial (RPE) cells to delay AMD, Omega-3 replaces the structurally similar Omega-6 [37], and also DHA induces cellular responses and mobilizes cellular resistance to misfolded proteins and oxidative stress [38,39,40]. DHA at doses of 700 mg daily has shown the most consistent benefits [41]. Xanthophylls lutein and zeaxanthin are oxygenated carotenoids exhibiting high antioxidant and anti-inflammatory potential. They can be found in different sources such as green vegetables, fruits, eggs, and microalgae, and they are delivered to the retina by plasma lipoproteins. In fact, lutein and zeaxanthin play a crucial role in vision health by protecting the eyes from harmful blue and ultraviolet light due to the formation of a protective pigment layer in the macular retina and RPE, thus maintaining the integrity of the retina [42,43,44]. There is increasing evidence supporting the hypothesis that the macular pigment carotenoids, lutein and zeaxanthin, play an important role in protection against AMD, by filtering out blue light or by quenching free radicals due to their antioxidant activity [45]. Table 2 and Table 3 show examples of natural drugs proposed for the prevention and treatment of AMD, including the major findings obtained in clinical trials and in animal experiments. 

There is strong evidence correlating the supplementation of lutein, zeaxanthin, and Omega-3 fatty acids with the reduction in AMD progression, therefore supporting these natural drugs as an alternative effective treatment for AMD patients. In fact, plenty of trials show the effectiveness of high clinical dosage of lutein and zeaxanthin complex (60 mg per day for 5 months) and Omega-3 PUFA (3.4 g of EPA and 1.6 g of DHA per for 6 months) on the significant improvement in vision acuity and the prevention of AMD progression in patients with both forms of early-AMD, wet and dry.

Another approach is the use of antioxidants, zinc, and carotenoids with DHA+EPA or without Omega-3 FA, in combination with anti-VEGF treatment, showing promising results and the association of Omega-3 FA in the reduction of vitreous VEGF-A levels in wet AMD patients. The effects of nutritional supplementation on AMD progression were evaluated by means of the Age-Related Eye Disease Studies (AREDS and AREDS2). These multicenter phase 3 randomized controlled trials were designed to assess the clinical course, prognosis, and risk factors of AMD and cataracts, and to also evaluate the effects of nutrients, including high-dose antioxidants, Omega-3 fatty acids, and zinc, on the progression of AMD and related vision loss. In the AREDS, 4757 participants (55–80 years of age) were recruited (from 1992–1998) and enrolled into AMD categories (no AMD to unilateral late AMD). In the AREDS2, 4203 participants (50–85 years of age) with bilateral large drusen or unilateral late AMD were recruited (from 2006–2008). However, it is important to remember that this is an older population and the study focused on more advanced stages of the disease (large bilateral drusen or large bilateral drusen in one eye and advanced AMD in the contralateral eye), and that the study design makes it difficult to observe large significant differences between groups (numerous small groups, comparative study). Other limitations of these supplements should be pointed out, AREDS 2 supplements cannot prevent early AMD from turning into intermediate AMD. It depends on what stage of age-related macular degeneration the patient is in. In intermediate AMD in one or both eyes, supplements may be able to prevent it from developing into advanced AMD. In advanced age-related macular degeneration in only one eye, AREDS 2 may slow the progression of macular degeneration in the other eye. If the patient already has advanced macular degeneration in both eyes, AREDS 2 supplements probably won’t help. On the other hand, some researchers have reported that some supplements for AMD available in the market do not contain the quantity of compounds described in the AREDS studies, which should be taken into account by patients and professionals. Different compositions of the formulations in the AREDS and ARESD2 assays are shown in Table 4.

AREDS2 improved the formulation proposed in the AREDS study by adding lutein and zeaxanthin and removing carotenes from the composition, as it was reported that carotenes inhibited the action of the other two compounds. Current and former smokers should take the AREDS2 formula and avoid the AREDS formula with beta-carotene because it increases the risk of lung cancer. Supplementation with AREDS2 vitamins can reduce the risk of progression to advanced disease by 25% and is recommended for patients with advanced AMD in one eye and early or intermediate disease in the fellow eye. Neither vitamin E nor total vitamin C consumption was associated with a statistically significant reduced risk for AMD. Among the specific carotenoids, lutein and zeaxanthin were most strongly associated with a reduced risk of AMD, whereas the intake of vitamin A (retinol) was not appreciably related to AMD [18], which was also confirmed in the VAST study, which concluded that lutein alone or in combination with other nutrients reduced the progression of AMD. Moreover, among patients with early AMD, supplementation with lutein and zeaxanthin improved macular pigment, which played a crucial role in visual function and might prevent the progression of AMD [43,46]. This supplementary diet has more interest in the prevention of late AMD, the potentially blinding stage of geographic atrophy (GA) and neovascular AMD. Neovascular AMD requires repeated intravitreal injection of endothelial growth factor drugs, whereas there are no clinically available treatments for GA either to slow down enlargement of the affected area or to restore vision loss. Agrón et al. (2021) [52] conducted research based on data from the AREDS and AREDS2 assays to examine potential associations between dietary intake of multiple nutrients and progression to late AMD, including separate analyses of GA and neovascular AMD. Preventative approaches based on a supplementary diet are attractive for late disease with the potential for blindness in different forms: neovascular AMD (requiring repeated intravitreal injection of endothelial growth factors) and geographic atrophy (GA) (for which no treatments are clinically available) [52]. Additional aims of this study included analyzing similar associations for the development of large drusen (using AREDS data) and examining potential interactions between nutrient intake levels and AMD genotype for the progression to late AMD. The results of this study show that for multiple nutrients (such as minerals, vitamins, and carotenoids), higher dietary intake is associated with a lower risk of progression to both subtypes of late AMD, but they are particularly strong for GA, for which no treatments are currently available. AREDS and AREDS2 supplements protect against neovascular AMD. Nutrients with protective associations against late AMD,, with a weaker level of evidence, also tend to have protective associations against the development of large drusen. On the other hand, strong genetic interactions exist for some nutrient genotype combinations, particularly between Omega-3 LC-PUFA intake and CFH genotype, which are involved in the progression to late AMD and GA.

Ongoing trials in AMD treatment propose the use of alternative antioxidant compounds that have gained attention, including flavonoids, curcumin, cerium oxide nanoparticles, quercetin, fucoidan, and co-enzyme Q10. However, further in vivo studies are needed to confirm the association of these compounds with an effective treatment of AMD disease. 

## 3. Biological Therapies for the Treatment of AMD

Regarding biological therapies, results of gen and stem cells therapy, currently under investigation, point them out as potential alternative treatments for AMD with a highly interesting prognosis [53,54,55]. 

Regarding gene therapy, there are some works proposing new alternative targets for designing new AMD treatment options and investigating genetic distribution of AMD-associated risk variants. However, the understanding of the genes and pathways involved in the early macular degeneration is still exploratory, there is an urgent medical need to identify the main associated genes. Recent studies on complement factor H (CFH) and the ARMS2/HTRA1 locus as the main genetic alterations associated in early disease manifestations resulted in some controversy due to the heterogenicity of the phenotype known as early AMD, meaning that not every early AMD manifestation in the form of large soft drusen, many small drusen, pigmentary changes in the RPE are caused by the same genetic risk factors [25,51]. On the contrary, the genes implicated in late AMD have been extensively studied. Main genetic variants identified are in ABCA4, variations in the apolipoprotein E (APOE) gene were also reported, GWAS, a common variant (rs1061170, p.Y402H) in the complement factor H (CFH), ARMS2/HTRA1 locus on chromosome 10 (10q26) [56,57,58,59,60].

AMD risk variants in the complement cascade were identified including complement component 3 (C3). On the other hand, there is considerable interest in using genetics as a tool for predicting the likelihood that a person will respond favorably (or unfavorably) to anti-VEGF therapy, suggesting that diagnostic and therapeutic strategies for AMD patients must include estimation of genetic interactions. Moreover, lifestyle was shown as a strong determinant of the outcome in each genetic risk category, with studies on the lifestyle factors of smoking and dietary intake of vegetables, fruit and fish. Current smokers with low intake of these foods showed at least a two-fold risk of late AMD, which is related to the natural drugs proposed for the prevention of AMD, as fruit, vegetables and fish are the main source of carotenoids and Omega-3 FA, respectively [61].

The genetic variants of rs5749482 (TIMP-3), rs11200638 (HTRA1), rs769449 (APOE) and rs6795735 (ADAMTS9) associated with AMD influenced the serum levels of IER3 (Immediate Early Response 3), HTRA1 (HtrA Serine Peptidase 1), and TIMP3 (tissue inhibitor of metalloproteinase 3) which may also be beneficial for the management of AMD patients. Moreover, different studies revealed that the genetic interaction between APOE-HTRA1 genotypes and changes in hepatic lipase levels play a crucial role in AMD. On the other hand, it has been suggested that patients who are homozygous for the variant risk C allele (CC genotype) in the polymorphism s1061170 (T1277C, Y402H) have a higher rate of persistent disease activity in comparison to homozygous for T-allele (TT genotype), whereas patients homozygous for the CFH Y402H risk allele required an increased number of injections of anti-VEGF drugs [57,62,63]. Additionally, experimental murine works have shown that the genetic deletion of IL-10 has also been shown to reduce capillary CNV size in the laser-induced CNV model as genetic deletion or pharmacologic inhibition of CaMKK2 (intermediate kinase calcium/calmodulin-dependent protein kinase 2), which is expressed in macrophages and amplifies circuit for signaling in immune cells in mouse models, diminishes inflammation. In addition, macrophage CaMKK2 is under evaluation as a target to reduce vision loss [64].

**Figure 2:**
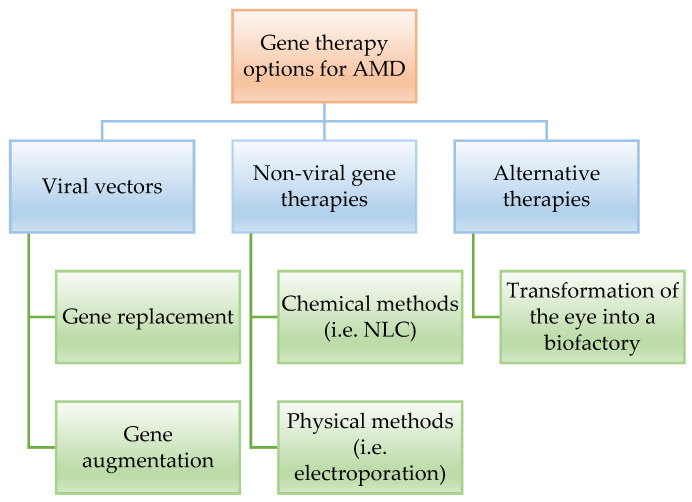
Schematic representation of the gene therapy options available in the literature showing their *Modus operandi*.

Regarding clinical gene therapy, the available options can be placed into three main alternatives (Figure 2). 

**Figure 3:**
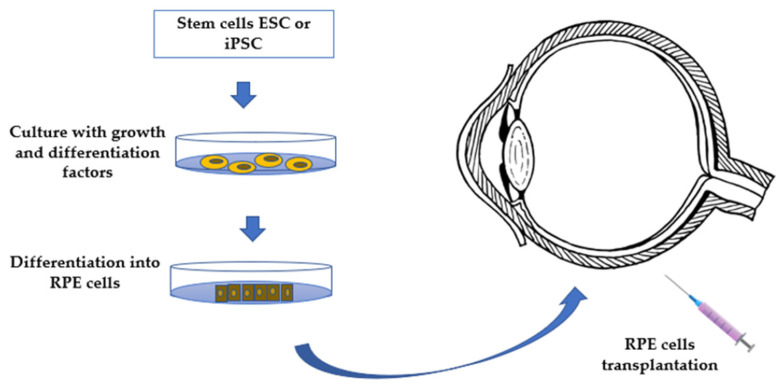
Stem cell therapy in AMD diagram [authors own drawing].

Naked plasmid DNA for gene transfer is ineffective because of limited cellular uptake, poor nuclear import, and rapid degradation by nucleases, resulting in low gene expression, thus, some alternatives are needed. Currently, viral vectors are the best option to replace the incorrect gene involved in the disease, with viral-mediated gene replacement therapies being the most studied. However, improved delivery systems must be implemented to ensure transduction efficiency and reduce iatrogenic risks. Indeed, apart from gene replacement therapy, the gene augmentation therapy seemed to be a promising alternative for non-inherent ocular disorders as AMD, ensuring the long-term sustained expression of secreted proteins in recent clinical trials. In fact, viral gene therapy has been explored as a treatment for both wet and dry AMD, with promising results. Nevertheless, the main future challenges of gene therapy still remain in the long-term expression and efficacy, despite the successful results in the literature [25,31,51,53,55,56,57,58,60,63,65,66,67,68,69]. Moreover, gene therapy has to overcome major economic challenges, taking into account the process from the manufacturing of the recombinant viral vectors, which includes still too low final yields. Indeed, the loading capacity of the vectors being too low is also a limiting point. Thus, major attention is put on the implementation of manufacturing and genetic engineering techniques to increase the yields and reduce the overall cost. The main non-viral gene therapies considered to overcome the disadvantages of viral therapies involve chemical and physical methods. Among the chemical methods, lipid and polymeric-based delivery systems have promising results in animal models with almost no-load limitation and are a potential alternative to viral gene therapies, especially for genes. However, the efficiency of DNA nanoparticles has not been yet proven in clinical trials. Regarding physical methods, we must highlight bio ballistic, electroporation, sonoporation, optoporation, and magnetofection, with electroporation being the most promising one, applying local and short external electric fields to the cells modifying the membrane permeability and allowing the plasmid with the DNA to penetrate. Electroporation following subretinal injection of naked plasmids resulted in efficient transduction of the neuroretina [58,65,68]. Some studies are currently ongoing using the gene therapy in order to transform the eye into an ocular biofactory to secrete anti-VEGF with promising results. Transduction of retinal cells to form an endogenous anti-VEGF biofactory is an attractive solution to decrease injection burden and improve durability. Currently, clinical trials are underway to explore gene delivery to natively express aflibercept, sFlt-1, and endostatin/angiostatin using different vectors, mainly AAV (adeno-associated virus), AAV.7m8, Lentiviral EIAV, AAV2 and AAV8, with indications on both, dry and wet, AMD by subretinal and intravitreal delivery [70]. In parallel, another important treatment option for AMD is the implantation of RPE cells from stem cells. Although it is also under investigation, there are two main approaches recently described. The most widely investigated involves the use of embryonic stem cells in RPE transplantation (ESC-derived RPE cells) which has been reported to be without any serious adverse outcomes on visual acuity, visual field, static perimetry, electroretinography or reading speed, and there were no signs of acute rejection, even after four years. The alternative therapy is based on induced pluripotent stem cells in RPE transplantation (iPSC derived RPE transplants) [33,57,68]. In fact, RPE cells can be differentiated from pluripotent stem cells and be transplanted subretinally to treat macular degeneration (Figure 3). However, this is not a universally ‘safe’ strategy. In fact, early attempts led to several enucleations of the treated eye, whereas cell transplantation can lead to proliferative vitreoretinopathy and retinal detachments, thus, there are still major problems to overcome. On the other hand, high potential tumorigenesis of iPSCs has become the biggest obstacle for clinic application and the tumorigenic genes in iPSCs have not been well documented yet. Ongoing investigations provide useful information for designing new strategies and methods to curtail the expression of oncogenic genes in iPSCs and produce safe derivatives for stem cell therapy, methods of pluripotency reprogramming that do not use genetic material provide another potential strategy for generating safe iPSCs. To date, various molecules that promote cell reprogramming have been reported as substitutes for genetic materials. Despite these promising results, nowadays, one of the major problems is the risk of reprogrammed stem cells developing into tumors, with this being the major obstacle to overcome [71,72,73].

Stem cells in the eye can perform two different functions as alternative therapies. The first one involves the regeneration or replacement of the RPE, whereas the second is a trophic role, in which they produce growth factors and cytokines, that have a supportive paracrine effect on local structures within the macula. Currently, there are two medium-term trials supporting the safety and survival of the cells transplanted and the effectiveness of the technique on the improvement of the macular degeneration up to a time period of 22 months [66,71,74,75]. Thus, long-term trials are still needed to assess the safety of the technique over the time, thus further studies needed to clarify safety and efficacy. There are key points in the stem cell therapy, as the conjunction of the RPE cells on a biodegradable support, such as collagen to improve the stability of the cells. Moreover, the environment before the transplantation should be treated to decrease the inflammatory and immune response and for the effectiveness of the technique, which could be overcome with the described natural drugs, such as the supplementation with Omega-3 lipids or lutein [32,37,40]. In fact, ESC-derived RPE cells combined with immunosuppression therapy showed effectiveness on the treatment of AMD patients. Despite these facts, stem cell therapy is shown as a promising alternative therapy to prevent the progression of macular disease, which is still being investigated in ongoing trials trying to establish the best conditions and the months of survival of the cells, determining the duration of the treatment. It must also be highlighted that most of the approaches to RPE transplantation use the intravitreal delivery method instead of the subretinal transplantation, which must also be further investigated [54]. 

Based on the exposed data, there is an urgent need to characterize the genetic alterations associated with AMD to increase the understanding of available treatment for this disease. Moreover, stem cell therapy seems to be an excellent candidate for AMD treatment with promising results on AMD models. However, stem cells are not authorized by FDA as a therapeutic alternative, thus, legal points must be overcome for the use of this alternative treatment. Nevertheless, despite the promising results that biological therapies show, these techniques have still not been fully explored yet, and further investigation is needed to better understand the factors involved in them. 

## 4. Drug Delivery Systems Proposed for AMD

Efficient ocular drug delivery to the posterior segment of the eye is a great challenge due to the special anatomy and physiology of the eye, making it difficult to achieve therapeutic drug levels in the internal eye structures following topical application [76,77]. The physical barriers of the eye (lacrimal barrier, corneal barrier, conjunctival barrier, clearance of aqueous humor circulation, and blood-retinal barrier (BRB)) become a great limitation for the transport of drugs and their passage to the retina [78]. On the other hand, certain drugs present problems for their use in ophthalmic therapy due to their solubility or their capacity to induce local or systemic side effects. Maximizing efficacy and minimizing adverse effects continues to be one of the main goals in ocular drug administration.

Drugs manage to cross the posterior segment of the eye through three non-invasive routes: (i) through the conjunctiva/sclera (topical application); (ii) cornea and aqueous humor (topical application); and (iii) via the systemic circulation (topical, parenteral or oral administration). Normally, the posterior segment is made up of tissues that are difficult to penetrate, for this reason different nanocarriers are being investigated in this field to overcome this barrier at the ocular level. The corneal epithelium is a membrane with large intracellular junctions, it is considered as a lipophilic barrier, where hydrophilic drugs can penetrate; but has resistance to lipophilic drugs [79]. Hydrophilic or low molecular weight drugs (less than 350 MW), cross this barrier through a paracellular pathway, while lipophilic drugs cross this barrier by 10% through the intracellular route. There are different factors that contribute to the absorption of drugs, among them we can name the physicochemical properties, integrity and formulation of the drug. The cornea acts as a reservoir for the drug, releasing it slowly through the aqueous humor, where drug levels gradually decrease; subsequently pass into the intraocular tissues. For this reason, a topical route is not adequate to achieve an ideal therapeutic concentration for the posterior segment [80].

Some reports recommend administering drugs intravitreally with targeted delivery of drugs to the RPE and thus successfully achieve the nanotherapies developed for the treatment of retinal diseases. Nanotherapies applied intravitreally to RPE cells are mainly based on the phagocytic nature of RPE, which phagocytizes endogenous materials, such as the outer segments of the photoreceptors and the exogenous material of the nanoparticles. Attempts have been made to direct the administration of drugs to the RPE with specific receptors such as VEGF, peptides sensitive to cathepsin D, systems are currently being investigated to be able to administer drugs specifically for the RPE [81].

There is a growing interest in the development of nanoparticles as drug delivery systems to the posterior chamber of the eye via topical administration because it is not invasive, overcoming the need for intravitreal injections, thus being able to deliver agents to the retina in a way other than via a syringe, with the additional advantage in delaying the release of drug and in reducing the risk of adverse effects. The inherent advantages of nanoparticles include increased surface area, variety of shapes, and surface chemical properties, including diameter, hydrophilicity, stability, porosity, and permeability [82].

Different kinds of nanoparticles decorated with functional ligands have been developed for retinal disease treatment. One of the important physicochemical characteristics is the particle size which influences the disposition through the ocular static and dynamic barriers and the extent of nanoparticles’ penetration [83]. Lipid nanoparticles, liposomes, polymeric nanoparticles, nanoemulsions, nanogels, nano-micelles, and dendrimers, are some examples proposed for retinal drug delivery.

**Figure 4:**
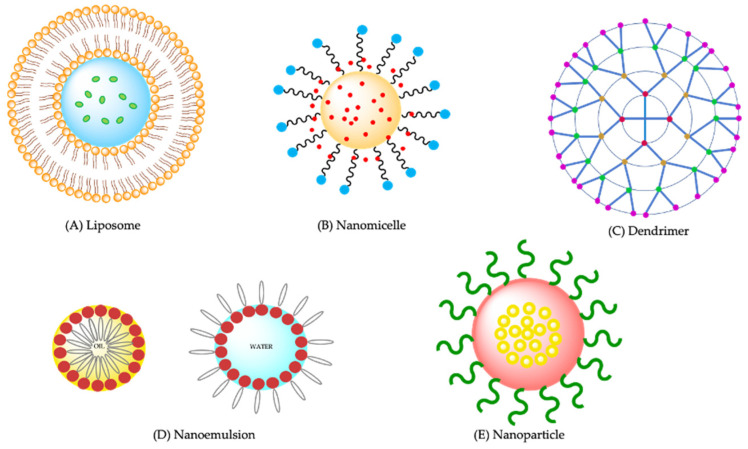
Schematic representation of common nanocarriers. (**A**) liposome; (**B**) Nanomicelle; (**C**) Dendrimer; (**D**) Nanoemulsion (oil-in-water type on the left-hand side; water-in-oil type on the right-hand side); (**E**) Nanoparticles (solid core surrounded by a surfactant layer). [authors own drawing].

Liposomes (Figure 4A) are advanced spherical vesicular drug delivery systems (DDS) that enclose an aqueous compartment surrounded by a phospholipid bilayer with a diameter in the range of 0.01–10 μm [84]. They have great similarity to biological membranes and excellent bioavailability, especially to the cornea and conjunctiva. In this way, the absorption and stability of the drug increased at this level and improves the prolonged retention time, without affecting optical properties or affecting vision. There are different liposomes taking into account the number of bilayers they present, which can be small unilamellar vesicles, large unilamellar vesicles and multilamellar vesicles, and may also contain between their layers specific functional molecules for a target organ where the drug must be released [85]. Various modifications can be made onto the surface of liposomes and drug release can be controlled by changing the composition of the lipid bilayer. Suri et al. (2020) [86] proposed to incorporate edge activators (polyol) in the liposomal bilayer in conventional liposomes that are inefficient to shuttle the cargo to the posterior segment of the eye to enhance the liposomal fluidity and deformability. In this way, polyol-modified liposomes would successfully penetrate the blood retinal barrier and would efficiently shuttle the drug sirolimus to the posterior segment of the eye making this a promising strategy for the treatment of posterior segment eye diseases. Karumanchi et al. (2018) [87] developed bevacizumab-loaded liposomes for extended released drug delivery to treat ocular angiogenesis, thereby decreasing the frequency and cost of treatment [87].

Nanomicelles are ordered amphiphilic structures, self-assembled in aqueous medium, with polar groups facing outwards and non-polar groups facing inwards, which facilities their use as a carrier for hydrophobic drugs (Figure 4B). Self-assembly occurs when the concentration is greater than the critical micelle concentration (CMC). Micelles are mostly 10 to 1000 nm in size. They have a hydrophobic core and a hydrophilic surface, mainly encapsulating hydrophobic drugs to increase their solubility [88]. Alshamrani et al. (2019) developed an aqueous nanomicellar drop formulation of curcumin for the delivery to the back of the eye, because the bioavailability of curcumin is negligible due to its poor aqueous solubility. The results showed that the nanomicelles offered improved protection against oxidative stress and significantly reduced vascular endothelial growth factor (VEGF) release in D407 cell line [89]. A tacrolimus nanomicellar formulation for AMD developed by Gote et al. (2020) showed that it could lower pro-inflammatory cytokines and the reactive oxygen species (ROS) commonly seen in this disease [90].

Dendrimers are well-defined macromolecules with a size ranging between 10 and 100 nm (Figure 4C). A focal symmetric core, a peripheral layer and an internal layer comprising of many building blocks constitute their structure [57]. They are non-immunogenic, stable, and capable of targeting specific targets [91]. In recent research, dendrimers have been combined with lipid systems. Lai et al. (2019) [92] designed a liposomal formulation that can entrap drugs using the third-generation polyamidoamine dendrimer (PAMAM G 3.0). These liposomes exhibited appreciable cellular permeability in human corneal epithelial cells and enhanced bio-adhesion on rabbit corneal epithelium and protective effects in human retinal pigment epithelial cells and rat retinas from photooxidative damage [92]. PAMAM-type dendrimers also act as a substrate for the synthesis of hydrogels, those that seal ophthalmological lesions and can produce cartilage tissue [91].

Nanoemulsions (Figure 4D) are transparent or translucent isotropic liquid-in-liquid dispersions of the water in oil (W/O) or oil in water (O/W) in the nanoscale range (with diameters of 1~100 nm) and can promote the efficient absorption of drugs into the tissue due to their good tissue permeability. Thus, improved patient compliance to treatment can be achieved, using smaller doses of medication and reducing side effects [78]. A novel penetratin-modified lutein nanoemulsion (P-NE) in-situ gel [63] was developed by Ge et al. (2020) [93] to improve the efficacy of lutein for AMD via a noninvasive strategy. The resultant system showed retinal cells protected from the photooxidative damage and eliminated ROS levels in cells. Therefore, the P-NE in-situ gel has a great potential for the treatment of AMD, particularly for dry AMD [93]. Laradji et al. (2021) [94] prepared a penetratin-complexed, redox-responsive hyaluronic acid-based nanogels to ensure the triggered release and, after topical application, increased delivery of actives to the posterior portion of the eye. They used as a model drug, visual chromophore analog, 9-cis-retinal, which was loaded into nanogels and efficiently delivered to the mouse retina’s photoreceptors when applied topically. This nanogel can deliver drugs to the back of the eye and has the potential in the treatment of AMD [94].

Nanoparticles (Figure 4E), one of the important carrier systems, are being widely used in nanomedicine, because they prolong the duration of drug release. First, the drug on the surface is released rapidly (burst effect), followed by a slower and more prolonged release of the drug contained within the nanoparticle. These systems can also increase the residence time in ocular tissues, they facilitate increased bioavailability, prolonged therapeutic effect, and decreased frequency of drug administration for effective treatment of chronic ocular diseases [76,95]. Among these, polymeric and lipid biodegradable nanoparticles have been proposed as a promising approach in the management of retinal diseases [96,97,98,99,100]. Different degradation rates and release kinetics can be achieved by modulating their surface chemistry (charge and hydrophilicity) and attachments, of nanoparticles [101]. Depending on the rate of release of the active from the nanoparticles, the frequency of administration can be modulated, avoiding intravitreal injections to allow patient compliance. 

Recent experiments were focused on demonstrating the role of atorvastatin [12] (statin, widely prescribed in the management of cardiovascular diseases), loaded to lipid nanoparticles (ATS-SLNs) for AMD treatment, due the fact that both diseases present similar pathological and risk factors [83]. ATS-SLNs, containing Compritol^®^ 888 ATO and Phospholipon 90H, prepared previously by a high-pressure homogenization method, were suitable to provide enhanced permeation across the cornea and attained higher drug bioavailability in both the aqueous and vitreous humor, avoiding side effects in other eye structures. Developed lipid NPs demonstrate safety in corneal and retinal cell lines which was confirmed by in vivo toxicity assays. The presence of ATS-SLN in the posterior segment of the eye, after topical administration, detected in fluorescence assays, constituted a new approach for the treatment of posterior eye disorders, including AMD and diabetic retinopathy. This and other recent studies on the development of nanoparticles being proposed to deliver drugs to the retina for the treatment of AMD are summarized in Table 5.

## 5. Three Dimensional (3D) Bioprinting Treatment for AMD 

Three dimensional (3D) bioprinting has high precision, reproducibility, and throughput. For this reason, it is necessary beforehand to make an adequate choice of bioinks, hydrogels and multicomponents, especially those that are characterized by being printable and biocompatible [102,103].

Currently, being able to build a functional 3D-printed human retina is a complex task. Every day more research is being carried out to achieve this goal through tissue engineering (TE), which promises in the future to repair the irreversibly damaged human retina and to restore its function. In this way, TE can overcome the inconveniences that occur in a tissue transplant, thus providing personalized treatment to patients. Studies by Lorber et al. (2016) showed that adult rat retinal ganglion and glial cells were successfully printed; in both cases, using piezoelectric inkjet technology [104]. Similarly, by 3D bioprinting technology, Shi et al. (2017) developed a retina of a monolayer of RPE (ARPE-19) and photoreceptors (Y79), where the printed construct can serve as a meaningful retinal model for in vitro investigations, with acceptable cytocompatibility that could reliably simulate a human retina [105]. In another study, Masaeli et al. (2020) reported the development of a specific retina for retinal regeneration therapies and a thin layer that simulated Bruch’s membrane, through 3D inkjet bioprinting, with successful results [106].

The application of 3D bioprinting can change the current and future perspectives of the treatment of patients with various ocular disorders, including AMD. Therefore, investigating more in this field in the future promises to be able to restore vision or reverse the advanced loss of RPE and photoreceptors, in those patients with AMD who do not respond to intravitreal anti-VEGF, implants or other types of conventional treatment [103].

Finally, it is considered important to monitor the progress of AMD treatment and disease. The most effective way is using retinal images. Optical Coherence Tomography (OCT) is the technique with the highest sensitivity and specificity to detect pseudodrusen [107,108,109]. Retinal fundus photography (FP) is a simple imaging technique, and is very useful for diagnosing both exudative and non-exudative AMD. When compared to OCT, FP has lower sensitivity to diagnosed CNV [110]. To detect wet AMD, the diagnosis with FP alone is considered inadequate because it underestimates the existence of CNV, therefore it is recommended to combine FP with another technique to avoid its limitations [111]. In comparison with FP, Fundus autofluorescence (FAF) has an advantage since it is possible to detect retinal alterations in the early and intermediate stages of AMD that may appear normal due to lower clarity in FP [112]. It is possible to identify the development of wet AMD with FAF with the identification of different patterns, i.e., patchy, reticular, and linear patterns. These techniques have the advantage of detecting the changes that can occur in the retinal tissues without an invasive procedure and avoiding systemic complications, while Fundus Fluorescence Angiography (FFA) and Indocyanine Green Angiography [91] are invasive procedures that require the injection of an intravenous contrast. IGA offers greater information on the location of CNV compared to FFA [2]. 

## 6. Conclusions

Anti-VEGF therapy is the standard treatment for AMD. The main disadvantage of anti-VEGF therapy is attributed to a number of consequences, such as intraocular inflammation, retinal detachment, and ocular hemorrhage occasionally due to the short half-life of the molecules, which require repeated intravitreal injections. Clinical trials are underway, focusing on stem cell therapy based on the transplantation of RPE cells for tissue damaged by choroidal neovascularization (CNV) or tissue loss owing to geographic atrophy. As for the natural drugs proposed for the treatment of AMD, most of them are based on the oxidative stress as the main therapeutic target for the management of the disease. Future strategies proposed for the prevention and treatment of AMD should focus on the suppression of oxidative stress and inflammation. To date, efficient ocular drug delivery to the posterior segment of the eye still poses a great challenge, thus, there is increasing interest in the development of formulations based on nanotechnology that could be useful because of the antecedents in managing posterior eye segment disorders, and due to the fact that drug delivery to the posterior chamber of the eye is less invasive, avoiding intravitreal injections, with the additional potential to reduce adverse effects and prolong drug release. In fact, based on the literature data, different kinds of nanocarriers have been developed for retinal drug delivery, from drug nanoparticles to nanoconstructs decorated with functional ligands for retinal disease treatment including lipid-based nanocarriers and liposomes, polymeric nanoparticles, nano or hydrogels nano-micelles or dendrimers, tested in cell and animal models. Similarly, there is a growing interest in developing studies with 3D bioprinting as an alternative to regeneration therapies for the human retina to restore its functions. The developed constructs, which simulate the retina and Brunch´s membrane, have acceptable cytocompatibility and can also serve as a model for in vitro investigations. In effect, the application of 3D bioprinting may change the current and future perspectives of the treatment of patients with ocular disorders, including AMD, especially those who do not respond to conventional treatment. Therefore, it is expected that new therapeutic targets for AMD, as well as more specific and less invasive diagnostic methods, will continue to be investigated in the following years.

**Table 5:** Selected preclinical studies based on novel drug-loaded biodegradable nanoparticles for the treatment of AMD.

Loaded Molecule	Matrix	Surfactant	AS	PDI	ZP (mV)	Assay Model	Results	Ref.
Atorvastatin	Compritol^®^ 888 ATO	PEG 400/Poloxamer 188	256.3 ± 10.5	0.26 ± 0.02	−2.65	Retinal pigment epithelial cells (ARPE-19)/rats	NPs were more bioavailable in the aqueous and vitreous humor than the free drug. Successful administration as eye drops was obtained in in vivo tests.	[83]
Resveratrol	PLGA	-	102.7 ± 2.8	0.095 ± 0.003	−47.30 ± 0.9	ARPE-19, human retinal pigment epithelial cell line (ATCC^®^ CRL2302^TM^)	The resveratrol-encapsulated PLGA nanoparticles were non-cytotoxic to the cells and exhibited effective inhibition of VEGF expression levels.	[113]
Sirolimus	PLGA (chitosan decorated)	PVA	265.9 ± 6.30	0.215 ± 0.12	+24.832 ± 2.12	Goat eyes/chick embryo chorioallantoic membrane [114]	The subconjunctival route has the greatest potential to surpass intravitreal injection as the preferred treatment option for AMD.	[115]
Lutein	PLGA (biotin-decorated)	PVA	208.0 ± 3.38	0.206 ± 0.016	27.2 ± 2.04	ARPE-19 cells	Biotin-conjugated nanoparticles may be an appropriate formulation for targeted drug delivery in the treatment of AMD and other retinal diseases	[116]
Axitinib	PLGA	PVA	131.33 ± 31.20	0.108 ± 0.005	−4.63 ± 0.76	ARPE-19, human retinal pigment epithelial cell line (ATCC^®^ CRL2302™)	This formulation may present an important approach to treating wet AMD due to its capability to ensure a sustained release of the drug and a potential in inhibiting VEGF expression.	[117]
DXM/Bevacizumab	PLGA/PEI	PVA	201.3 ± 7.2	0.318 ± 0.084	0.31 ± 1.15	Human umbilical vein endothelial cells (HUVECs)/chick embryo chorioallantoic membrane	The formulation provided a strong inhibitory effect on VEGF secretion from HUVECs. Moreover, in vivo chick embryo chorioallantoic membrane assay showed nanoparticles greatly reduced the amount of blood vessels.	[118]
Doxorubicin	PEG-PLA chain with Tat-C (CPP)	-	29.4 ± 4.1	-	−1.0 ± 0.2	Human umbilical vein endothelial cells (HUVECs)/adult female C57BL/6 mice	Doxorubicin-loaded NPs-CPP significantly reduces neovascular lesion size, proposing a strategy for non-invasive treatment of CNV, and enhancing drug accumulation specifically in diseased areas of the eye.	[119]
Fenofibrate	PLGA	PVA	265 ± 10	0.03 ± 0.01	−1.2 ± 0.1	Male Brown Norway and Vldlr^−/−^ mice	Ameliorated retinal dysfunctions, reduced retinal vascular leakage, inhibited retinal leukostasis, and downregulation of the overexpression of VEGF were observed. Furthermore, Fenofibrate NPs reduced retinal vascular leakage and CNV formation in both animal models.	[120]

AS: average size; CNV: choroidal neovascularization; CPP: cell penetrating peptide; DXM: dexamethasone; NPs: nanoparticles; PEG: polyethylene glycol; PDI: polydispersity index; PEI: polyethylenimine; PLA: poly(lactic acid) PLGA: poly(lactic-*co*-glycolic acid); PVA: poly(vinyl alcohol); VEGF: vascular endothelial growth factor; ZP: zeta potential.

## Figures and Tables

**Table 1:** Anti-VEGF agents proposed for AMD treatment in clinic.

Drug	Clinical Dose for AMD (mg)	Structure	MOA	Limitations	FDA Approval
Ranibizumab^®^	0.5	Fab fragment	Anti–VEGF-A	Time intervals between injections lower than Faricimab^®^.	2006
Bevacizumab^®^	1.25	Full antibody (IgG1)	Anti–VEGF-A	Not approved by FDA. Higher dose required than Ranibizumab^®^.	Not approved
Aflibercept^®^	2.0	VEGFR1/2-Fc fusion protein	Anti–VEGF-A/PIGF/VEGF-B	Lower safety and efficacy than Brolucizumab^®^.	2011
Ziv-Aflibercept^®^	1.25	VEGFR1/2-Fc fusion protein	Anti–VEGF-A/PIGF/VEGF-B	Not approved by FDA Higher dose required than Ranibizumab^®^.	Not approved
Conbercept^®^	0.5	VEGFR1/2-Fc fusion protein	Anti–VEGF-A/VEGF-B/VEGF-C/and PIGF	Not approved by FDA Higher dose required than Ranibizumab^®^.	Not approved
Brolucizumab^®^	6.0	scFv	Anti–VEGF-A	Higher dose required than Ranibizumab^®^.	2019
Faricimab^®^	6.0	Angiopoietin-2	Anti–VEGF-A	Higher dose required than Ranibizumab^®^.	2022

FDA: Food and Drug Administration; MOA: mode of action; nAMD: neovascular age-related macular degeneration; PIGF: placental growth factor; VEGF: vascular endothelial growth factor; IgG1: immunoglobulin G1; scFv: single-chain variable fragment; VEGFR: vascular endothelial growth factor receptor.

**Table 2:** Natural drugs proposed for AMD prevention in clinical trial studies on early AMD patients.

Drug	MOA	Retinal Findings	Limitations	References
Lutein, β-carotene and zeaxanthin	Protection of RPE from oxidative stress.	- Lower oxidative stress was found in the retina. - Supplementation with lutein and zeaxanthin improved macular pigment. - Visual function was improved with lutein alone or lutein together with other nutrients. - ARM was significantly associated with combined plasma lutein and zeaxanthin. - Protective role of the xanthophylls, in particular zeaxanthin, for the protection against ARM and cataracts.	In large doses, β-carotene can cause lung and gastric cancer in smokers. This is the reason why it isn’t recommended in these patients.	[42,43,44,46,47,48,49]
Antioxidant supplement containing lutein, vitamins A, C and E	Antioxidant activity, Protection of RPE from oxidative stress.	Lutein and zeaxanthin were the most strongly associated with AMD.	No effectiveness in advanced macular degeneration in both eyes.	[18]
EPA and DHA	Antiangiogenic and anti-inflammatory effects, protecting the retina against oxidative stress.	Significant improvement in vision acuity occurred was observed in AMD patients after Omega-3 supplementation.	More clinical studies are needed to confirm its effectiveness.	[34,35,36,38,39,40]
Omega-3 + anti-VEFG	VEGF limited via anti-VEGF molecules, antiangiogenic and anti-inflammatory effects, lowering oxidative stress in the retina.	Omega-3 supplementation combined with anti-VEGF treatment was associated with decreased vitreal VEGF-A levels in wet AMD patients.	More clinical studies are needed to confirm its effectiveness.	[27]

AMD: age-related macular degeneration; ARM: age-related maculopathy; DHA: Docosahexaenoic acid; EPA: eicosapentaenoic acid; MOA: mode of action; RPE: retinal pigment epithelium; VEGF: vascular endothelial growth factor.

**Table 3:** In vitro and in vivo studies of natural drugs proposed for early AMD prevention.

Drug	MOA	Retinal Findings	Limitations	References
Cerium oxide nanoparticles	Simulation of the activity of superoxide dismutase and catalase preventing retinal function loss and preserving retinal morphology.	- Nanoceria incorporated into an alginate-gelatine based injectable hydrogel was effective in cellular models of AMD. - Glycol chitosan-coated cerium oxide nanoparticle promoted RPE and photoreceptor recovery by reducing proinflammatory mediators and cellular apoptosis in Nrf2 knockout mice exposed to mild, white light.	Further evaluation is needed beyond these in vitro studies.	[25,31,32,50]
Curcumin	Regulation of the expression of oxidative stress biomarkers and apoptosis-associated proteins such as AKT, Nrf2, cytokines, and NF-kB.	Treatment with curcumin can regulate oxidative stress and apoptosis in in vitro studies.	Further studies are needed to fully assess curcumin’s potential as an antioxidant therapy for early AMD.	[31,32]
Fucoidan	Reduction of the expression of VEGF in RPE and choroidal cells that could decrease angiogenesis. Downregulation hypoxia inducible factor-1a/VEGF and PI3K/Akt signaling.	Promising results in in vitro studies. Further evaluation is needed for treating early AMD.	Further evaluation is needed beyond these in vitro studies.	[31,32]
Flavonoids	Inhibition of NF-kB and activation of AP-1 and Nrf2. Regulation of IL-6/JAK2/ STAT3 signaling pathway in RPE cells, which has been implicated in AMD pathobiology.	- Eriodictyol, quercetin, luteolin, and taxifolin have antioxidant effects. - Eriodictyol is the most potent and efficient antioxidant in cultured retinal cells.	Further evaluation is needed.	[31,32]
Quercetin	Activation of the intracellular redox system including Nrf2 signaling, the caveolin-1 pathway, proinflammatory cytokines, and apoptosis.	Inhibition of a VEGF-induced inflammatory response through MAPK/Akt signaling, NF-kB translocation in mouse RPE cells, and the formation of choroidal neovascularization in both in vitro and in vivo models of AMD treatment of both dry and wet AMD.	Further evaluation is needed beyond these in vitro studies.	[31,32]
Coenzyme Q10	Essential component of the electron transport chain necessary for respiration with ROS-scavenging ability	- Exogenous Co-Q10 induced cellular reductases improving the cellular oxidative stress status. - Restoration of endothelial cell function, which may improve retinal and visual function and protect neuroretinal cells from oxidative stress. - Low levels of Co-Q10 have been found in patients with AMD.	Further evaluation is needed.	[23,31,32,51]

AMD: age-related macular degeneration; RPE: retinal pigment epithelium; VEGF: vascular endothelium growth factor; ROS: reactive oxygen species; MAPK: mitogen-activated protein kinase; Akt: protein kinase B; NF-kB: NF-kappaB, “nuclear factor kappa-light-chain-enhancer of activated B cells”; Nrf2: Nuclear factor-erythroid factor 2-related factor 2; PI3K: phosphatidylinositol 3-kinases; IL: interleukin; STAT3: Signal transducer and activator of transcription 3; JAK2: Janus kinase 2.

**Table 4:** Commercially available formulas based on AREDS and AREDS2.

Nutrient	AREDS Formula *	AREDS2 Formula
Vitamin C	500 mg	500 mg
Vitamin E	400 IU	400 IU
Beta-carotene	15 mg	-
Copper (cupric oxide) **	2 mg	2 mg
Lutein	-	10 mg
Zeaxanthin	-	2 mg
Zinc	80 mg	80 mg

* Not recommended for current or former smokers; ** Added to avoid zinc-related copper deficiency; mg: milligrams; IU: international units.

## Data Availability

Not applicable.
